# Social independence evaluation index for Japanese patients with childhood-onset chronic diseases

**DOI:** 10.3389/fped.2022.923497

**Published:** 2022-12-06

**Authors:** Yuzaburo Inoue, Hiroaki Umebayashi, Toshihiro Matsui, Susumu Nishiyama, Ikuho Sakurai, Mitsue Maru, Tetsuro Takeda, Koji Tanigawa, Takako Miyamae

**Affiliations:** ^1^ Department of General Medical Science, Graduate School of Medicine, Chiba University, Chiba, Japan; ^2^ Department of Pediatrics, Eastern Chiba Medical Center, Togane, Japan; ^3^ Department of Allergy and Rheumatology, Chiba Children's Hospital, Chiba, Japan; ^4^ Department of General Pediatrics, Miyagi Children's Hospital, Sendai, Japan; ^5^ Department of Rheumatology Research, Clinical Research Center for Allergy and Rheumatology, National Hospital Organization Sagamihara National Hospital, Sagamihara, Japan; ^6^ Rheumatic Disease Center, Kurashiki Medical Center, Kurashiki, Japan; ^7^ Department of Nursing, Faculty of Health Sciences, Saitama Prefectural University, Koshigaya, Japan; ^8^ Child Nursing, College of Nursing Art and Science, University of Hyogo, Akashi, Japan; ^9^ Faculty of Education, Wakayama University, Wakayama, Japan; ^10^ Department of Teacher Education, Kobe Shoin Women's University, Kobe, Japan; ^11^ Pediatric Rheumatology, Institute of Rheumatology, Tokyo Women's Medical University, Tokyo, Japan

**Keywords:** social independence, activity, transitional care, international classification of functioning, disability and health (ICF), social participation (MeSH), childhood-onset, chronic diseases, delphi technique [MeSH]

## Abstract

**Introduction:**

This study established an independent evaluation index for patients with childhood-onset chronic diseases in Japan.

**Methods:**

From November to December 2020, three Delphi rounds were conducted. Thirty-nine participants completed at least one survey. We asked them about targets of social independence for 10 types of activities (education/labor/finance/acquisition of necessities/housing/transportation/leisure/social relationship/intimate relationships/sexuality). The Delphi technique was to be repeated until a consensus of over 80% of participants was reached.

**Results:**

The targets chosen for measuring independence in patients with childhood-onset chronic diseases were as follows: “Graduation from high school,” “Labor for livelihood (including temporary turnover),” “Financially independent (including temporary turnover, excluding students),” “Buy or rent a house and buy the daily necessities and get the public services you need to live,” “Do housework alone,” “Plan alone and use transportation to get around,” “Participate in play/recreation/leisure activities on own initiative,” “Engage in relationships with other people outside of a limited environment (home, school, office, hospital, etc.),” “Create and maintain intimate or romantic relationships between individuals (couples, lovers, sexual partners),” and “Use or know how to use contraceptives and how to prevent sexually transmitted diseases.”

**Conclusions:**

We established an independent evaluation index for patients with childhood-onset chronic diseases in Japan through a three-round Delphi process. The assessment of social independence using our independent evaluation index may help plan for and provide appropriate support and assistance to these patients.

## Introduction

The transition from pediatric to adult healthcare systems has recently garnered attention worldwide (1). Various interventions have been used to improve the transition process and have been reported to improve outcomes in population health, experience of care, and utilization and cost of care (2, 3). However, most previous studies have focused on outcomes of transitional care in patients with specific diseases (2, 3).

The goals of transitional care is reportedly “to maximize lifelong functioning and potential through the provision of high-quality, developmentally appropriate healthcare services that continue uninterrupted as the individual moves from adolescence to adulthood (4).” Thus, one goal of transitional care is to improve the quality of social life when the patient reaches adulthood.

Individuals have increased opportunities to engage in various activities and participate in society as they mature from adolescence to adulthood. Furthermore, a notable shift has been observed from the dependency of childhood to greater independence in a range of social roles (5). However, there is some concern that children and adolescents with special healthcare needs, even with appropriate medical care, may have problems finding employment and building relationships, as they cannot engage in the events and activities necessary for social independence.

Therefore, in this study, we focused on social independence, which is one of the outcomes of transitional care, to establish an independent evaluation index for patients with various types of childhood-onset chronic diseases in Japan, with reference to the Rotterdam Transition Profile (RTP) used for cerebral palsy patients (6).

## Materials and methods

The study was approved by the ethics review board of Chiba Children's Hospital, Chiba, Japan (Approval number, 2019-021).

Initially, the steering committee of the study was organized. The committee consisted of seven members of the transitional care committee of the Pediatric Rheumatology Association of Japan (three pediatric rheumatologists, two non-pediatric rheumatologists, and two transitional care nurses) and two education professionals who specialized in teaching children with chronic diseases.

The items of the RTP was based on the contents of “activity and participation” of the International Classification of Functioning (ICF), Disability, and Health (7). Thus, we also referenced the ICF and made an original draft of targets of social independence for 10 types of activities (education/labor/finance/acquisition of necessities/housing/transportation/leisure/social relationship/intimate relationships/sexuality) appropriate for or expected of adult patients with childhood-onset chronic diseases in Japan (Table 1). Furthermore, in line with the ICF philosophy, we decided to evaluate the subjects’ performance of activities and participation as well as their capacity to engage in these activities while receiving any support/assistance.

**Table 1:** Original draft and voting results in the three Delphi rounds.

Items	Draft	Leader in the 1st round	Leader in the 2nd round	Leader in the 3rd round
		Leading percentage (%)	Leading percentage (%)	Leading percentage (%)
Education	Enrolled in or graduated from a vocational school or university/professional education institution	Draft	Graduation from high school or vocational school	• Graduation from high school or vocational school • Graduation from high school
		48.7	32.4	50.0 each
Labor	Labor for livelihood (including temporary turnover)	Draft		
		89.7		
Finance	Financially independent (including temporary turnover)	Draft	Financially independent (including temporary turnover, excluding students)	Financially independent (including temporary turnover, excluding students)
		71.8	55.9	90.0
Acquisition of necessities	Buy or rent a house, buy the daily necessities you need, and get the public services you need to live	Draft		
		87.2		
Housing	Do housework alone	Draft		
		89.7		
Transportation	Plan alone and travel using transportation	Draft		
		82.1		
Leisure	Plan and carry outplay, recreation, and leisure activities	Draft	Participate in play/recreation/leisure activities on own initiative	Participate in play/recreation/leisure activities on own initiative
		74.4	26.5	96.7
Social relationship	Join relationships with other people outside a limited environment (home, school, office, hospital, etc.)	Draft		
		87.2		
Intimate relationships	Create and maintain intimate or romantic relationships between individuals (couples, lovers, sexual partners)	Draft		
		87.2		
Sexuality	Experienced with sexual intercourse or able to use contraceptives and prevent sexually transmitted diseases	Draft	“Educated about sexual intercourse” or “Can use contraception and prevent sexually transmitted diseases”	“Educated about sexual intercourse” or “Can use contraception and prevent sexually transmitted diseases”
		59.0	26.5	63.3

From November to December 2020, three Delphi rounds were conducted. A Delphi consensus survey was performed using an online tool (Google Forms). The survey was disseminated to leading pediatric physicians, leading non-pediatric physicians, transitional care nurses, and members of patient/guardian groups in rheumatology, endocrinology, nephrology, allergology, and cardiology. None of the participants received financial compensation.

The participants were informed that the patients' diseases targeted by this project were not associated with any intellectual disability or severe physical disability that would prevent engaging in social activities, even with appropriate support/assistance. The Delphi technique was to be repeated until a consensus of over 80% of participants was reached.

## Results

Thirty-nine participants completed at least one survey. Table 2 presents the participant characteristics.

**Table 2:** Participant characteristics.

	Pediatric physician (*n*)	Non-pediatric physician (*n*)	Pediatric Nursing (*n*)	Non-pediatric Nursing (*n*)	Patient/guardian groups (*n*)
Rheumatology	3	2	1	1	2
Cardiology	2	1	1	1	2
Nephrology	2	1	1	1	1
Endocrinology	1	1	1	1	0
Gastroenterology	1	1	1	1	0
Allergology	1	1	2	0	1
Transitional care	2	0	2	0	0

We received responses from 39 participants for Round 1. Participants either expressed agreement with the original draft or—if they disagreed with any point—were asked to present a counter-proposal for each item of social independence. In 6 of the 10 items, more than 80% of the participants agreed with the contents of the draft text. Consequently, the draft text was adopted for inclusion in the social independence evaluation index.

In Round 2, we presented the original draft and all counter-proposals for the remaining four items and asked the participants to select one of each item. We received responses from 34 participants. In “Finance” and “Entrainment,” only one answer received more than 20% but not 80% of votes. We therefore asked the participants whether they agreed to adopt the answers in Round 3. In “Education” and “Sexuality,” two answers received more than 20% but not 80% of votes. We therefore asked the participants to select one of them in Round 3.

In Round 3, we received responses from 30 participants. In “Finance” and “Entrainment”, the leading answers received ≥80% of votes, but “Education” or “Sexuality” did not receive more than 80% of the votes.

Finally, the members of the steering committee discussed the independent evaluation index for “Education” and “Sexuality.” As the last two proposals included “graduation from high school,” we concluded that “graduation from high school” might be a minimum requirement for independence. In terms of “Sexuality,” it tends to be difficult to discuss an individual's experience in sexual intercourse in clinical practice. We therefore focused on contraceptives and the prevention of sexually transmitted diseases. We finally decided that the independent evaluation index for “Sexuality” in Japan in this study would be “Using or understanding how to use contraceptives and how to prevent sexually transmitted diseases.”

## Discussion

This study established a consensus concerning independent evaluation indices for patients with childhood-onset chronic diseases in Japan using a three-round Delphi process. The ability to participate in certain social roles and activities considered to indicate “independence” differs among countries, and people in different positions have different perceptions concerning this ability. We therefore recruited not only medical doctors but also transitional care nurses and members of patient/guardian groups to discuss the social situation in Japan.

Our independent evaluation index differs from the RTP established in the Netherlands (6) in several respects (Table 3). We divided “Education and Employment” in RTP into two items of “Education” and “Labor” because a higher education does not necessarily lead to a higher income, and the support systems that encourage independence differs between these two items. Similarly, we divided “Leisure (social activities)” in RTP into two items of “Leisure” and “Social relationship,” as social relationships in non-leisure situations, such as at school or in the office, are also considered important for achieving independence. In addition, we added “Acquisition of necessities,” an item included in “activity and participation” of the ICF (7) and considered important for people with disabilities.

**Table 3:** A comparison of the independent evaluation index established in this study and the RTP.

	Final consensus on the independent evaluation index in this study	RTP
Education	Graduation from high school	Paid job, volunteer work
Labor	Labor for livelihood (including temporary turnover)	
Finance	Financially independent (including temporary turnover, excluding students)	Economically independent: job income, benefit
Acquisition of necessities	Buy or rent a house, buy the daily necessities you need, and get the public services you need to live	
Housing	Do housework alone	Living independently
Transportation	Plan alone and use transportation to get around	Young adult arranges transportation for themselves
Leisure	Participate in play/recreation/leisure activities on own initiative	Young adult goes out in the evening with peers
Social relationship	Join relationships with other people outside of a limited environment (home, school, office, hospital, etc.)	
Intimate relationships	Create and maintain intimate or romantic relationships between individuals (couples, lovers, sexual partners)	Young adult has a current romantic relationship/a partner
Sexuality	Use or know how to use contraceptives and prevent sexually transmitted diseases	Young adult has experience with sexual intercourse

RTP, rotterdam transition profile.

Education is essential for learning self-dependence. Education in high school in Japan is considered upper secondary education in the International Standard Classification of Education 2011 (8). This level is defined as the second and final stage of secondary education, preparing for tertiary education, and providing skills relevant to employment. In Japan, the admission rate to high school was 95.8% in 2019, and financial support and special schools were available for students with special educational needs owing to learning difficulties, physical disabilities, or behavioral problems.

Labor force participation is linked with higher rates of social inclusion (9). Therefore, regardless of whether financial independence is achieved, labor is considered an essential part of participation in society.

In addition, being economically independent is also very important. However, patients with chronic diseases have lower incomes than healthy individuals (10). Therefore, future studies should focus on income generation methods, given the declining income of young individuals and the uncertainty concerning social welfare in Japan.

“Acquisition of necessities,” “Housing,” and “Transportation” are important skills for daily life. We noted no significant difference in the content between the RTP and our independent evaluation index.

Building relationships is essential in life. In both “Leisure” and “Social relationship,” building relationships—even with unfamiliar people—is necessary for independence. This is leads to more social activity outside the limited environment (home, school, work, etc.). Interestingly, the independent evaluation index for “Intimate relationships” in this study was similar to that in the RTP, while that for “Sexuality” focused less on sexual relationships than that for the RTP, probably due to differences in customs between countries.

At present, opinions vary regarding the most effective implementation methods of the index in clinical practice, including the age at which the evaluation should be started and the frequency of performing such evaluations. In particular, there are conflicting opinions regarding whether it is acceptable and appropriate to conduct evaluations by including topics such as “Intimate relationships” and “Sexuality” from an early age. A preliminary study is therefore considered to be necessary to determine the most appropriate timing to conduct such evaluations.

Nevertheless, the present study had some limitations. First, because of the SARS-CoV-2 pandemic, we were unable to meet in person to discuss and vote on the Delphi rounds. Thus, we voted *via* the web tool, which might have affected the results of this study. Second, we did not consider the influence of disease-specific disabilities in this study. Therefore, future studies should explore whether the results of this study could be applied to actual individual diseases. Third, because the social environment differs among countries, the events and activities required for social independence in patients with chronic diseases might also differ.

In conclusion, we have developed a non-disease-specific independence evaluation index for patients with childhood-onset chronic diseases in Japan through a three-round Delphi process. We believe that the assessment of social independence using the index can be used to support pediatric patients with chronic diseases, and assess patients with different chronic diseases.

## Data Availability

The raw data supporting the conclusions of this article will be made available by the authors, without undue reservation.
